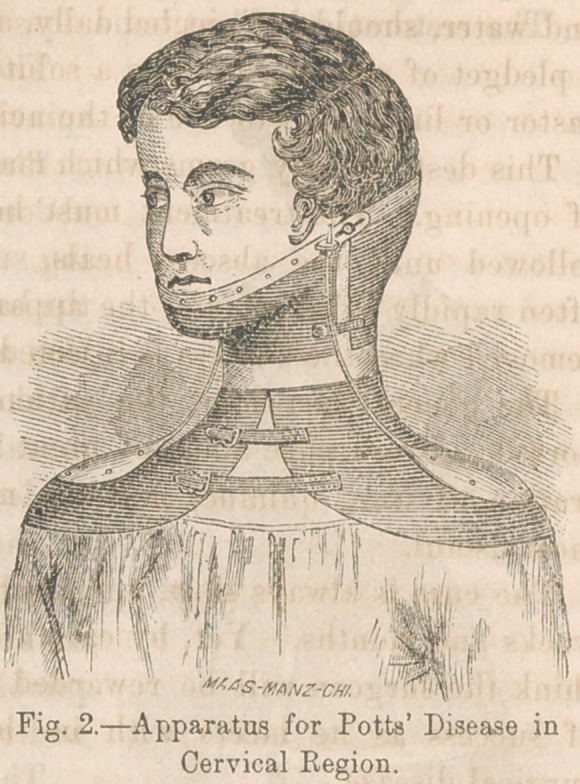# Backward Curvature of the Spine

**Published:** 1869-10

**Authors:** J. S. Sherman

**Affiliations:** Lecturer on Orthopœdic Surgery in the Chicago Medical College; 81 Monroe Street


ARTICLE XXXVII.

### BACKWARD CURVATURE OF THE SPINE.

### ITS PATHOLOGY AND TREATMENT.

By J. S. SHERMAN, M.D., Lecturer on Orthopœdic Surgery in the Chicago Medical College.

The neglect of surgical authors treating of diseases of the spine, must be plainly visible to any one examining the subject from the text-books of the present day, and much more evident in those of older authority.

A few general remarks on their appearance, cause, and treatment is all the information given, and the practitioners with a case under his observation, turns from the books with little or no idea as to the best course to pursue with his patient. An apparatus of some kind is recommended, which lifts from the axilla, to remove weight from the spine. Bringing pressure on the axillary nerves, where it cannot be tolerated by any child or adult. When we consider that the shoulders are freely moveable, upwards and downwards, to a very considerable extent, it is evident that they must be raised as high as the ears, before they begin to relieve any weight from the bodies of the vertebræ. Yet these concerns are often fastened upon the backs of patients, with the seeming idea, that there is something curative in the simple contact of them. The relief of pressure from the inflamed bones, is by no means accomplished. In addition to the mechanical treatment, the seaton and irritating applications, upon and in the neighborhood of the spine, are still advised. The origin is generally referred to scrofula or tuberculosis, and said to occur mostly in the children of the poor, and badly fed, being one of the diseases most frequently found in poverty and rags. From this doctrine and treatment, I think we have the best of reasons to dissent.

Those treating the disease most extensively, do not refer its origin to a deposit of tubercle. Nor do they most frequently meet with it among those deprived of the necessaries of life.

The strong and robust are as often its victims as the weak and sickly. Taylor claims that the former are more often its subjects.

Adams, one of the surgeons to the Royal Orthopœdic Hospital of London, told me that in his practice he had seen the disease as frequently in patients without the hospital as within.

My own observation coincides with that stated, and I have been able, in the majority of all cases, to trace the point of departure directly to an injury. It is not strange that the backs of children are injured by slight falls, or by twisting the spine. The cartilagenous attachments of the five centres of ossification in each vertebræ, become consolidated only in the adult. In early life an inflammation is easily awakened in this tissue by violence. I have recorded in this city three cases of the disease occurring in adults, all strong and healthy. The first: a man aged thirty years, by trade a painter, fell from a scaffold and was treated for a sprain of the back; he partially recovered and was again able to do light work. Four months after, a decided projection of the tenth dorsal vetebræ was noticed, with all the characteristics and symptoms of "Potts'" disease.

The second, a laborer, aged thirty-five, was injured by falling from a car. Three months after the injury, projection of the sixth and seventh dorsal vertebræ occurred.

The third, a physician, presented symptoms of inflammation of the vertebræ, six months after a slight injury of the back.

I cite these cases to show that the disease occurs from injury of the spine, in the adult as well as the child; that it excites an inflammation, simple in its character, at first involving the cartilages only, but in time producing the backward curvature.

The tubercular constitution is not necessary, and in a majority of cases neither exists, nor is it developed. We have to contend with a simple inflammation, but one occurring in structures surrounded by conditions which will not allow of spontaneous recovery. These conditions are, constant pressure and movement on the inflamed surfaces.

The treatment consists in relieving pressure and securing immobility. We are not able in the spine, as in the joints, to make direct extension, but overcome pressure upon the bodies of the vertebræ, by supining the back and bringing the line of gravity through the articulating processes, which are not involved in inflammation. This is the object of all mechanical appliances and those are useless which do not accomplish it. Confining the patient to bed should be avoided as much as possible, as such a course impairs the general health.

Various forms of apparatus have been constructed by different surgeons. From experience in the use of all, I find none which so well fulfils the indications for treatment, as that figured in cut No. 1. This accomplishes its purpose so effectually, and is tolerated with such ease and comfort to the patient, that I have used no other for the last year.

In children it forms a support for the back, which they can wear night and day, and in which they can be carried about with ease and safety.

Often, in the early stages, the inflammation extends to the sheath of the spinal cord and is prolonged over the coverings of the nerves, deriving their origin from the diseased point. This frequently causes paralysis of the structures supplied by them. It always somewhat impairs their function. Pain, when present, is referred to the termination of these nerves. Disease in the cervical region impairs the power of speech, and sometimes the memory. In the dorsal, apparent gastric trouble and disturbance of respiration. In the lumbar region, pains in the bowels, with a diminution of control over the legs.

Occurring in any portion of the column, the patient makes an effort to supine the back and avoid any jar in walking. The positions which are taken show a desire to transfer the weight from the bodies to the articulating processes.

The diagnosis can generally be made from these symptoms *before* any projection is seen. It is important to recognize the condition early, as we may then prevent deformity, and restore to the patient a spine perfect in its symmetry.

After the disease has advanced to the loss of substance of the bodies and projection of the spinous processes, the diagnosis is no longer a matter of doubt, and we can then only hope to save life and produce anchylosis of the vertebræ in their deformed position.

The restoration of lost bone does not occur.

Fig. 1.—Apparatus for Backward Curvature of the Spine.

The mode of constructing the spinal support is as follows:

The patient is placed upon the abdomen, the chest and hips being supported by pillows, so that the back is supined. In this position a mould in plaster of Paris of the back, hips, and shoulders is taken. From this a cast is made which gives an exact model of the back, with the column supined. A piece of sole leather, soaked in water, until soft and pliable is lashed upon the cast, and remains until dry.

The front is completed with a corset and axillary straps. A few strips of steel are riveted to the backpiece to prevent its form changing. When adjusted the back is secured in the supined position, the bowels supported by the corset, and the line of gravity thrown through the articulating processes.

Absolute immobility is secured. The relief to the patient is decided and immediate. When the disease occurs in the upper cervical vertebræ, it is not so easy to remove pressure. In these cases the weight of the head must be *directly* removed from the inflamed bones.

We can here use the shoulders as a support for an apparatus. Those forms by which the head is secured with a chin and occipital strap, cannot be tolerated when sufficient tension is made on them to support the weight. The accuracy with which we can adapt leather to a cast, and the evenness with which it distributes the pressure when applied to the body, has led me to construct an apparatus for the relief of these cases, in the same manner as for those occurring in other regions of the spine.

Figure 2, represents the instrument. A cast is taken, reaching from the shoulders as high as the mouth in front, the ears on the side, and the bulge of the occipital bone behind. Leather is then moulded upon this as in the construction of the first apparatus.

Fig. 2.—Apparatus for Potts' Disease in Cervical Region.

From either shoulder rises a rod upon which is cut a screw. The chin and occipital piece are divided laterally and the upper margin of each surrounded with a steel strip. The posterior half having upon each side a slot, into which the steel of the chinpiece slides, and is secured with a thumbscrew.

This allows us to easily apply the head piece, and to grasp with any amount of firmness. The screw rods, provided with a nut, pass through a ring attached to the steel band surrounding the headpiece.

By turning the nuts we are enabled to lift the entire weight of the head from the cervical vertebræ. The inner surface is lined with soft buck-skin, and no inconvenience is complained of by the patients wearing it.

TREATMENT OF ABSCESS.

When the inflammation has progressed to caries and suppuration, the abscess may point either in the groin or upon the back.

As soon as we are confident of its nature, the contents should be evacuated.

The controlling effect of carbolic acid over suppuration bids fair to render the treatment of these abscesses much more satisfactory than formerly. A saturated solution of carbolic acid and water, should be injected daily, and the opening sealed with a pledget of cotton dipped in a solution of eight parts of either castor or linseed oil to one of the acid.

This destroys any germs which may have entered at the time of opening. The treatment must be effectually and diligently followed until the abscess heals. The pain and tenderness often rapidly subsides, and the apparatus can be worn and only removed when the abscess is injected.

The general health of the patient should be carefully supported. Fresh pure air is of great importance. The administration of iron, quinine, and the mineral acids will be found most useful.

The cure is always slow, and improvement follows only after weeks and months. Yet, by careful watching of these cases, I think the surgeon will be rewarded with as large a proportion of success as he meets with in the treatment of any other surgical disease.

*81 Monroe Street.*

## Figures and Tables

**Fig. 1. f1:**
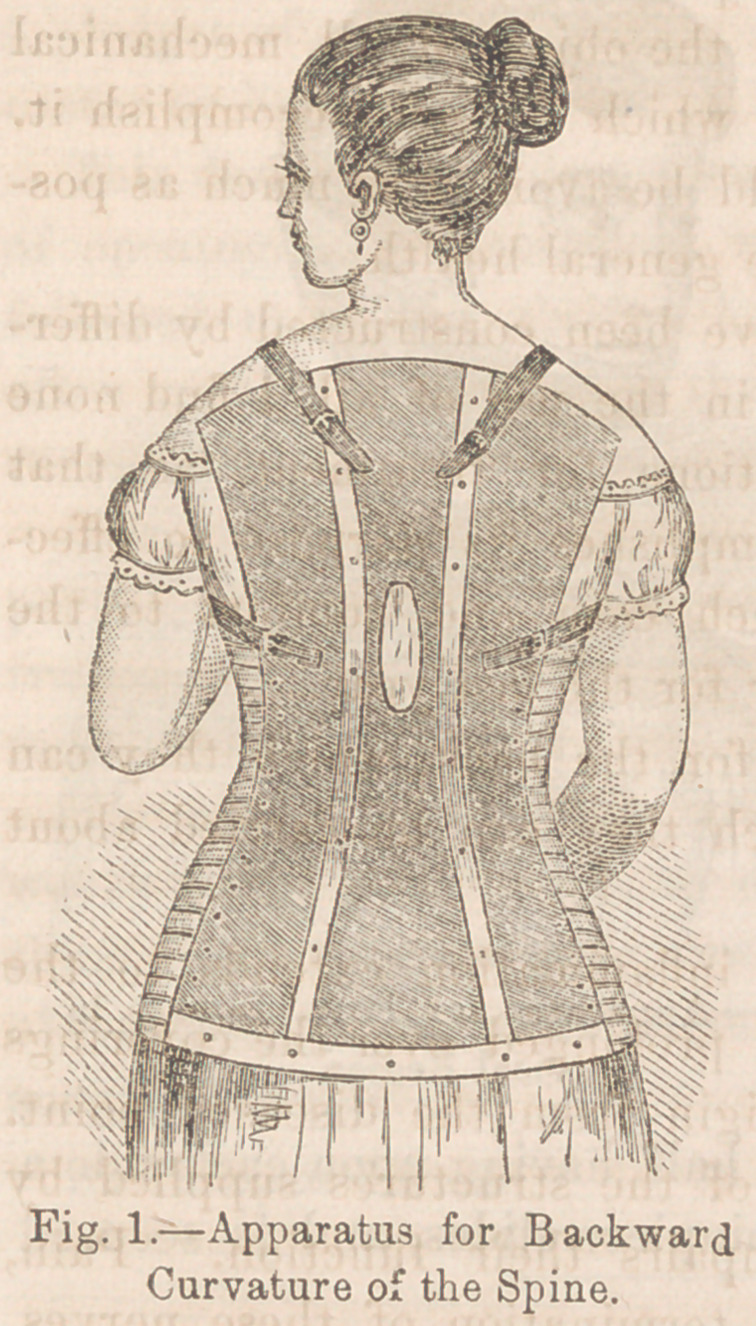


**Fig. 2. f2:**